# Labour market marginalisation in young adults diagnosed with attention-deficit hyperactivity disorder (ADHD): a population-based longitudinal cohort study in Sweden

**DOI:** 10.1017/S0033291721002701

**Published:** 2023-03

**Authors:** Magnus Helgesson, Emma Björkenstam, Syed Rahman, Klas Gustafsson, Heidi Taipale, Antti Tanskanen, Lisa Ekselius, Ellenor Mittendorfer-Rutz

**Affiliations:** 1 Department of Clinical Neuroscience, Division of Insurance Medicine, Karolinska Institutet, SE-17177 Stockholm, Sweden; 2 Department of Neuroscience, Psychiatry, Uppsala University, Uppsala, Sweden; 3 Department of Global Public Health, Karolinska Institutet, Stockholm, Sweden; 4 Niuvanniemi Hospital, Kuopio, Finland; 5 School of Pharmacy, University of Eastern Finland, Kuopio, Finland

**Keywords:** ADHD, anxiety disorder, attention deficit hyperactivity disorder, depression, disability pension, labour market marginalisation, sick leave, stress disorder, unemployment

## Abstract

**Background:**

The objective of this population-based register study was (1) to investigate the association between young adults diagnosed with attention-deficit/hyperactivity disorder (ADHD) and subsequent labour market marginalisation (LMM) in two comparison groups, i.e. matched young adults from the general population without ADHD and unaffected siblings to persons with ADHD and (2) to assess the role of comorbid disorders.

**Methods:**

This study included all young adults in Sweden, aged 19–29 years, with an incident diagnosis of ADHD 2006–2011 (*n* = 9718). Crude and multivariate sex-stratified hazard ratios (HRs) with 95% confidence intervals (CIs) were measured 5 years after the diagnosis of ADHD for the risk of disability pension, long-term sickness absence (SA) (>90 days), long-term unemployment (>180 days) and a combined measure of all three in young adults with ADHD compared to their siblings without ADHD and a matched comparison group.

**Results:**

In the adjusted analyses young adults with ADHD had a 10-fold higher risk of disability pension (HR = 10.2; CI 9.3–11.2), a nearly three-fold higher risk of long-term SA (HR = 2.7; CI 2.5–2.8) and a 70% higher risk of long-term unemployment (HR = 1.7; CI 1.6–1.8) compared to the matched comparison group. The risk estimates were lower compared to siblings for disability pension (HR = 9.0; CI 6.6–12.3) and long-term SA (HR = 2.5; CI 2.1–3.1) but higher in the long-term unemployed (HR = 1.9; CI 1.6–2.1). Comorbid disorders explained about one-third of the association between ADHD and disability pension, but less regarding SA and long-term unemployment.

**Conclusions:**

Young adults with ADHD have a high risk for different measures of LMM and comorbidities explain only a small proportion of this relationship.

## Introduction

During the past decades, there has been an increased awareness that attention-deficit hyperactivity disorder (ADHD) is not only affecting children as previously believed, but that symptoms of inattention might also persist into adulthood. In a study including 10 countries on three continents, about 3.5% of individuals in working-age were found to have symptoms of ADHD (de Graaf et al., 2008). Because ADHD is characterised by attention deficiency, lack of impulse control and problems controlling activity level, the disorder may lead to significant negative consequences affecting the individual's ability to work (Helgesson, Tinghog, Niederkrotenthaler, Saboonchi, & Mittendorfer-Rutz, 2017; Hirvikoski, Lindström, Nordin, Jonsson, & Bölte, 2017; Wiklund, Patzelt, & Dimov, 2016). Furthermore, the number of young adults who have been diagnosed with ADHD in young adult age has increased markedly since 2000 (Edvinsson, 2017; Giacobini, Medin, Ahnemark, Russo, & Carlqvist, 2018; Rydell, Lundström, Gillberg, Lichtenstein, & Larsson, 2018; Thomas, Sanders, Doust, Beller, & Glasziou, 2015). Consequently, labour market marginalisation (LMM), i.e. severe problems in obtaining and keeping a job, could be a serious problem for young adults with ADHD. Because young adults have most of their working life ahead of them, early LMM may lead to long-term productivity loss. However, the most significant impact is on the individual who will likely risk economic hardships and potentially further deteriorating health (Janlert & Hammarstrom, 2009; McKee-Ryan, Song, Wanberg, & Kinicki, 2005). Still, to date information on the magnitude of LMM in young adults is lacking. Hence, it seems warranted to conduct a tailor-made design intervention for this patient group.

The scope of LMM can only be assessed by including information on unemployment, which most existing studies have done (de Graaf et al., 2008; Kupper et al., 2012) and on work disability, i.e. sickness absence (SA) and disability pension. Many of the individuals diagnosed with ADHD might never enter the labour market. Thus, there is a risk of underestimating the actual rate of marginalisation in young adults with ADHD (Helgesson et al., 2017). Because Sweden´s welfare system has several measures of LMM, which often act as communicating vessels, a combined measure of LMM which allows comparison to other countries is desirable. Moreover, few studies have assessed the consequences of ADHD in young adults and hence the working-age population. Most of these studies are conducted on self-reported data using a cross-sectional design, which might result in underestimation of both the prevalence of ADHD and the consequences (i.e. LMM) (de Graaf et al., 2008; Kupper et al., 2012). It is, therefore, vital to measure the scope of marginalisation in this patient group using population-based studies with longitudinal data of high quality.

Commonly, young adults with ADHD suffer from comorbidities (e.g. depression, anxiety, substance use, asthma, diabetes mellitus and stress-related and autism-spectrum disorders) (Aduen et al., 2018; Bjorkenstam, Pierce, Bjorkenstam, Dalman, & Kosidou, 2020; Chen, Lee, Yeh, & Lin, 2013; Cortese et al., 2018; Edvinsson, Lindstrom, Bingefors, Lewander, & Ekselius, 2013; Kupper et al., 2012). These comorbidities may further negatively affect occupational functioning and increase the difficulties of obtaining and retaining a job. Still, detailed studies of the role of a full range of different comorbid diagnoses regarding the association between ADHD and subsequent LMM are not available. Moreover, studies have reported large sex differences in the prevalence of ADHD (Edvinsson et al., 2013). However, there are also differences in the symptomatic profile, where women more often have internalising problems, whereas men tend to have more externalising problems (Edvinsson et al., 2013; Gershon & Gershon, 2002). Given that the risk of LMM also differs by sex, analyses stratifying for sex are needed in related studies.

Other socio-demographic factors also influence the association between ADHD and LMM. For instance, educational attainment seems to be lower in young adults with ADHD compared to the general population (Kupper et al., 2012). In Sweden and other Scandinavian countries, education level is an important determinant of LMM, as most jobs demand at least upper secondary education (Nilsson, 2017). Moreover, other socio-demographic factors (e.g. civil status, number of children at home, type of living area and work-related factors, such as previous labour market integration) may be relevant in the association between ADHD and labour market integration (Giacobini et al., 2018). Finally, concerning ethnicity, migrants seem to have a higher prevalence of ADHD compared to the host population (Lehti, Chudal, Suominen, Gissler, & Sourander, 2016). It is therefore essential to consider all these socio-demographic factors in the data analyses regarding LMM. In addition to the characteristics mentioned above, psychosocial determinants during childhood and adolescence, as well as family-related and genetic factors, may influence the future risk of LMM in young adults with ADHD (Svedberg et al., 2011). For this reason, the present study compared the risk of LMM in young adult patients with ADHD to the unaffected general population and the patients' siblings without ADHD, who share familial and genetic factors, including predisposition for certain disorders.

This population-based longitudinal register study aimed (1) to assess the risk of LMM − measured as long-term unemployment, long-term SA and disability pension − and a combined measure of these three outcome measures, in young adult men and women with incident ADHD and compare it to comparison groups consisting of persons in the general population without ADHD and unaffected siblings to persons diagnosed with ADHD and (2) to investigate the role of comorbid disorders in these relationships.

## Methods

### Registers

Data from five registers were merged individually based on the de-identified unique personal identification number given to all permanent residents in Sweden. Information was available for each individual retrospectively from 1 January 2005 and prospectively until 31 December 2016 from the following five Swedish nationwide registers: (1) *Longitudinal Integration Database for Health Insurance and Labour Market Studies* (*LISA*), hosted by Statistics Sweden: all socio-demographic variables, year of emigration and unemployment; (2) *Microdata for Analysis of Social Security* (*MIDAS*) hosted by the Swedish Social Insurance Agency: date, duration and grade of SA and disability pension; (3) the *Multi-Generation Register* (*MGR*), hosted by Statistics Sweden, with information on parents and siblings to persons with ADHD; (4) the *National Patient Register* (*NPR*): primary and secondary diagnoses for ADHD and all comorbid disorders during the year of the cohort entry date (CED, 2006–2011); and (5) *Cause of Death Register*: date of mortality (2005–2016). Databases 4 and 5 are hosted by the Swedish National Board of Health and Welfare.

### Study population

The study base was made up of 16 647 young male and female adults between 19 and 29 years of age (mean age = 23.4 years) who had the first primary or secondary diagnosis of ADHD from either inpatient or specialised outpatient health care. This information was derived from the NPR between 2006 and 2011. The year of the incident diagnosis served as the CED. Individuals who had an ongoing disability pension (*n* = 4727) at the CED or a record of ADHD medication before the CED (*n* = 2202) were excluded. The final study population comprised of 9718 young adults with ADHD.

We defined two comparison groups from the five registers. For one of the comparison groups, we selected five individuals from the general population without ADHD matched for sex, age and educational level) with no ongoing disability pension (*n* = 48 590). The second comparison group consisted of siblings not affected by ADHD in the same age range (19–29 years) as their siblings with ADHD and who were not on disability pension (*n* = 5582). Siblings were matched on both mother and father, i.e. only full siblings were considered in the analysis. In the case of two (or more) siblings with ADHD who were close in age, the person with the ADHD diagnosis and the earliest cohort entry date was chosen as the exposed individual.

### Variables

#### Exposure variable

ADHD was defined based on the diagnostic code (F90) of the International Classification of Diseases, 10th Revision (ICD-10).

#### Outcome measures

The cohort was followed for 5 years from the CED (2006–2016) for (1) disability pension, (2) long-term SA (>90 annual net days of SA registered at the Swedish Social Insurance Agency), (3) long-term unemployment (>180 annual days registered as full-time unemployed at the Swedish Public Employment Service) and (4) total LMM, defined as granting either the disability pension, long-term SA or long-term unemployment.

#### Covariates

Covariates in the analyses included (1) *socio-demographic factors* (sex, age, educational level, family composition, type of living area and country of birth), all measured on 31 December the year before CED, (2) *work-related factors* (unemployment and SA, both measured during the year before CED) and (3) *comorbid disorders* (information about the primary and secondary diagnoses of inpatient and specialised outpatient health care in 2005–2011 due to depression and bipolar disorders (ICD-10: F30-F34), anxiety- and stress-related disorders (ICD-10: F40-F48), autism-spectrum disorders (ICD-10: F84), substance use (ICD-10: F10-F19), behavioural and emotional disorders (ICD-10: F91-F98), schizophrenia/non-affective psychoses (ICD-10: F20-F29), mental retardation/disorders of psychological development (ICD-10: F70-F83, F85-F89), other mental disorders (ICD-10: Other F codes), musculoskeletal disorders (ICD-10: M01-M99), asthma (ICD-10: J45), diabetes mellitus (ICD-10: E10-E14), neoplasms (ICD-10: C00-D48), cardiovascular disorders (ICD-10: I00-I99), accidents (ICD-10: S00-S99) and other somatic disorders (ICD-10: remaining codes for somatic disorders except O.80 and Z00–99). The categorisation of all covariates is presented in Table 1.

**Table 1.** Characteristics of patients with ADHD, diagnosed in specialised health care in 2006–2011 (*N* = 9718) and individuals without ADHD (general population *N* = 48 590, matched for sex, age and educational level and the patients' siblings *N* = 5582) (number (*n*) and per cent (%) distribution)

	General population		Siblings	
	Individuals with ADHD	General population	Individuals with ADHD	Siblings
Total, *n* (%)	9718 (16.7)	48 590 (83.3)	4382 (44.0)	5582 (56.0)
Sex				
Female	4166 (42.9)	20 830 (42.9)	1866 (42.6)	2813 (50.4)
Male	5552 (57.1)	27 760 (57.1)	2516 (57.4)	2769 (49.6)
Age				
19–24 years	6037 (62.1)	30 185 (62.1)	2818 (64.3)	3364 (60.3)
25–29 years	3681 (37.9)	18 405 (37.9)	1564 (35.7)	2218 (39.7)
Educational level (years)				
Elementary school (<10)	4665 (48.0)	23 325 (48.0)	1985 (45.3)	1203 (21.6)
Upper secondary school (10–12)	4221 (43.3)	21 055 (43.3)	1965 (44.8)	3174 (56.9)
University (>12)	842 (8.7)	4210 (8.7)	432 (9.9)	1205 (21.6)
Region of birth				
Sweden	8954 (92.1)	38 560 (79.4)	4180 (95.4)	5244 (93.9)
Other Nordic countries	73 (0.8)	501 (0.8)	18 (0.4)	24 (0.4)
EU27	100 (1.0)	1014 (2.1)	10 (0.2)	18 (0.3)
Rest of world	591 (6.1)	8615 (17.7)	174 (5.0)	296 (5.3)
Family composition^a^				
Married/cohabiting without children	116 (1.2)	1520 (3.1)	46 (1.0)	131 (2.3)
Married/cohabiting with children	744 (7.7)	6808 (14.0)	328 (7.5)	741 (13.3)
Single without children	8318 (85.6)	38 699 (79.6)	3793 (86.6)	4492 (80.5)
Single with children	540 (5.6)	1565 (3.2)	215 (4.9)	218 (3.9)
Type of living area				
Big cities	3329 (33.4)	18 661 (38.4)	1465 (33.4)	2009 (36.0)
Medium-sized cities	3567 (36.7)	17 682 (36.4)	1614 (36.8)	2031 (36.4)
Small towns	2822 (29.0)	12 247 (25.2)	1303 (29.7)	1542 (27.6)
Unemployment at baseline				
0 days	5728 (58.9)	35 763 (73.6)	2577 (58.8)	4064 (72.8)
1–180 days	3419 (35.2)	11 041 (22.7)	1551 (35.4)	1314 (23.5)
>180 days	571 (5.9)	1786 (3.7)	254 (5.8)	204 (3.7)
SA at baseline				
0 days	8142 (83.8)	46 096 (94.9)	3653 (83.4)	5210 (93.3)
1–90 days	660 (6.8)	1809 (3.7)	312 (7.1)	261 (4.7)
>90 days	916 (9.4)	685 (1.4)	417 (9.5)	111 (2)
Comorbidity (ICD-10-code^b^ in parentheses)				
Mental disorders				
Anxiety- and stress-related disorders (F40-48)	2154 (22.2)	927 (1.9)	991 (22.6)	165 (3.0)
Autism-spectrum disorders (F84)	144 (1.5)	35 (0.1)	73 (1.7)	< 10
Behavioural and emotional disorders (F91-98)	224 (2.3)	29 (0.1)	106 (2.4)	< 10
Depression and bipolar disorders (F30-34)	1688 (17.4)	696 (1.4)	802 (18.3)	126 (2.3)
Eating disorder (F50)	145 (1.5)	71 (0.2)	66 (1.5)	19 (0.3)
Mental retardation^c^ (F70-83, F85-89)	59 (0.6)	18 (0.1)	30 (0.7)	< 10
Schizophrenia/psychoses (F20-F29)	129 (1.3)	75 (0.2)	69 (1.6)	< 10
Substance use (F10-19)	1706 (17.6)	761 (1.6)	780 (17.8)	120 (2.2)
Other mental disorders (all remaining F-codes)	909 (9.4)	295 (0.6)	405 (9.2)	51 (0.9)
Somatic disorders				
Accidents (S00-S99)	1263 (13.0)	3181 (6.6)	570 (13.0)	395 (7.1)
Asthma (J45)	100 (1.0)	187 (0.4)	48 (1.1)	33 (0.6)
Cardiovascular disorders (I00-I99)	112 (1.2)	300 (0.6)	46 (1.0)	37 (0.7)
Diabetes mellitus (E10-E14)	96 (1.0)	362 (0.8)	47 (1.1)	59 (1.1)
Musculoskeletal disorders (M01-M99)	490 (5.0)	1492 (3.1)	235 (5.4)	187 (3.4)
Neoplasms (C00-D48)	122 (1.3)	551 (1.1)	52 (1.2)	79 (1.4)
Other somatic disorders (all remaining codes^d^)	3475 (35.8)	10 246 (21.1)	1544 (35.2)	1304 (23.4)

a With children living at home.

b International Classification of Diseases, Version 10.

c Including disorders of psychological development.

d Except for ICD-10 codes O80 (single delivery) and Z00-Z99 (factors influencing health status and contact with health services).

### The Swedish social insurance regulations

Individuals ⩾16 years of age can receive sickness benefits if they have an income from an established business or employment. During the first 14 days, except for the first day, which is a qualifying day, the employer covers these sickness benefits. From day 15, the Swedish Social Insurance Agency then takes over to continue to pay the benefits, and from that day, data are available in registers. Individuals between 19 and 29 years of age can receive a time-restricted disability pension when their work capacity is reduced or if compulsory education is not completed at the age of 19. Persons from 30 years of age can only be granted permanent disability pension. Individuals >16 years of age can be enrolled at the Swedish Public Employment Service where they can receive unemployment benefits. From age 20, basic levels of unemployment benefits can also be received without earlier income from work.

### Statistical analyses

Cox proportional hazard regression models with competing risks (cause-specific hazards) were applied to calculate hazard ratios (HRs) with 95% confidence intervals (CIs) in order to determine the association between ADHD and subsequent outcomes of LMM (Andersen, Geskus, de Witte, & Putter, 2012; Koller, Raatz, Steyerberg, & Wolbers, 2012). Emigration and mortality were regarded as competing events in all the models. In the analyses regarding both long-term SA and long-term unemployment, also disability pension was seen as a competing event. The follow-up period was 5 years, starting from 1 January in the year following the incident diagnosis of ADHD (CED). All analyses were conducted in three steps: (1) A crude model, (2) Model 1, adjusted for sex, age, educational level (by matching with the general population), family composition, type of living area and country of birth, (3) Model 2, like model 1 and additionally adjusted for unemployment and SA in the year before the CED and (4) Model 3, like model 2 and additionally adjusted for all comorbid disorders.

For the sibling analyses, conditional Cox proportional hazard regression models with competing risks (cause-specific hazards) were performed to adjust for shared familial confounders, i.e. genetic factors and unmeasured shared confounders such as socioeconomic status, neighbourhood or stable parental factors. To assess the contribution of specific comorbid conditions a set of analyses was conducted. Here, individuals with specific comorbid disorders were excluded one at a time, with the remaining individuals compared to the matched comparison group. All analyses were carried out using SAS, version 9.4 (SAS Institute Inc.).

## Results

Periods of unemployment, as well as SA in the year before the CED, were much more prevalent in young adults diagnosed with ADHD than in both comparison groups (Table 1). Moreover, young adults with ADHD were less often married and more likely to live in small towns compared to young adults without ADHD. The most striking finding, however, was that the prevalence of common mental comorbidity was nearly 10 times higher, and the prevalence of most comorbid somatic disorders was twice as common in young adults with ADHD than in the comparison group without ADHD. The most commonly occurring mental comorbid disorders were depressive, anxiety and substance use disorders.

In total, 21% of the young adults with ADHD were granted disability pension within 5 years after the incident diagnosis, which can be compared to just 2% in both the matched comparison group (Table 2) and the patients' siblings without ADHD (Table 3). In the crude model young adults diagnosed with ADHD had more than a 15-fold higher risk of disability pension (HR = 15.59, Table 2) compared to the matched comparison group. The risk estimates were slightly decreased when adjusting for socio-demographic factors (6% lower risk estimate), and they did not decrease when adjusting for work-related factors, however, they decreased substantially when adjusting for comorbid disorders (an additional 29% lower risk estimate). In the final model young adults with ADHD still had over 10 times higher risk of receiving disability pension (HR = 10.2) compared to the matched comparison group (Table 2). The adjusted risk estimates were comparable between adult women and men and somewhat lower when compared to the comparison group of siblings (HR = 8.9, Table 3).

**Table 2.** HRs with 95% CIs for LMM, measured as disability pension, long-term SA (>90 days) and long-term unemployment (>180 days) in persons with diagnosed attention-deficit hyperactivity disorder (ADHD) (*n* = 9718) compared to a matched cohort of individuals without ADHD (*n* = 48 590)

			Crude model	Model 1^a^	Model 2^b^	Model 3^c^
		*n* (%)	HR (95% CI)	HR (95% CI)	HR (95% CI)	HR (95% CI)
Disability pension						
All	General population	749 (2)	1	1	1	1
	ADHD	2075 (21)	15.59 (14.34–16.95)	14.66 (13.46–15.96)	14.53 (13.34–15.84)	10.18 (9.27–11.19)
Women	General population	349 (2)	1	1	1	1
	ADHD	1038 (25)	17.01 (15.07–19.20)	15.75 (13.91–17.83)	15.54 (13.71–17.60)	11.06 (9.64–12.68)
Men	General population	400 (1)	1	1	1	1
	ADHD	1037 (19)	14.42 (12.85–16.18)	13.84 (12.31–15.56)	13.71 (12.17–15.43)	9.65 (8.47–10.98)
Long-term SA						
All	General population	3498 (7)	1	1	1	1
	ADHD	2336 (24)	4.27 (4.05–4.50)	4.32 (4.09–4.55)	3.24 (3.07–3.43)	2.67 (2.51–2.84)
Women	General population	2044 (10)	1	1	1	1
	ADHD	1174 (28)	3.82 (3.56–4.11)	3.78 (3.51–4.07)	2.94 (2.72–3.18)	2.45 (2.25–2.68)
Men	General population	1454 (5)	1	1	1	1
	ADHD	1162 (21)	4.96 (4.59–5.36)	5.11 (4.72–5.53)	3.68 (3.38–4.00)	3.03 (2.76–3.32)
Long-term unemployment						
All	General population	6423 (13)	1	1	1	1
	ADHD	1840 (19)	1.71 (1.62–1.80)	2.03 (1.92–2.14)	1.76 (1.67–1.86)	1.65 (1.55–1.75)
Women	General population	2471 (12)	1	1	1	1
	ADHD	595 (14)	1.43 (1.31–1.57)	1.79 (1.63–1.97)	1.65 (1.50–1.82)	1.55 (1.40–1.73)
Men	General population	3952 (14)	1	1	1	1
	ADHD	1245 (22)	1.88 (1.76–2.00)	2.18 (2.04–2.32)	1.83 (1.71–1.96)	1.68 (1.56–1.81)
Total LMM						
All	General population	9948 (20)	1	1	1	1
	ADHD	5243 (54)	3.57 (3.46–3.70)	3.94 (3.81–4.08)	3.27 (3.15–3.38)	2.69 (2.59–2.80)
Women	General population	4516 (22)	1	1	1	1
	ADHD	2336 (56)	3.58 (3.41–3.77)	3.96 (3.76–4.17)	3.37 (3.20–3.55)	2.78 (2.62–2.96)
Men	General population	5432 (20)	1	1	1	1
	ADHD	2907 (52)	3.57 (3.41–3.73)	3.95 (3.77–4.13)	3.21 (3.06–3.36)	2.63 (2.49–2.77)

a Adjusted for sex, age and educational level by matching, as well as for family composition, type of living area and region of birth.

b As Model 1 and additionally adjusted for baseline unemployment and baseline SA.

c As Model 2 and additionally adjusted for comorbidities for mental and somatic disorders.

**Table 3.** HRs with 95% CIs for disability pension, long-term SA, long-term unemployment and total LMM in patients with diagnosed ADHD (*n* = 4382) compared to their siblings without ADHD (*n* = 5582)

		Patients with ADHD	Crude model	Model 1^a^	Model 2^b^	Model 3^c^
		*n* (%)	HR (95% CI)	HR (95% CI)	HR (95% CI)	HR (95% CI)
Disability pension	Siblings	99 (2)	1	1	1	1
	ADHD	902 (21)	13.92 (11.06–17.50)	11.84 (9.22–15.21)	11.86 (9.22–15.25)	9.00 (6.61–12.27)
Long-term SA	Siblings	517 (9)	1	1	1	1
	ADHD	1042 (24)	3.42 (3.03–3.86)	4.20 (3.63–4.86)	3.06 (2.61–3.59)	2.54 (2.11–3.05)
Long-term unemployment	Siblings	611 (11)	1	1	1	1
	ADHD	861 (20)	2.27 (2.02–2.55)	2.03 (1.79–2.30)	1.92 (1.68–2.19)	1.85 (1.60–2.14)
Total LMM	Siblings	1128 (20)	1	1	1	1
	ADHD	2340 (53)	3.79 (3.48–4.12)	3.77 (3.43–4.14)	3.23 (2.93–3.57)	2.77 (2.48–3.10)

a Adjusted for sex, age, educational level, family composition, urban area and region of birth.

b As Model 1 and additionally adjusted for baseline unemployment and baseline SA.

c As Model 2 and additionally adjusted for comorbidities for mental and somatic disorders.

Some 24% of individuals with ADHD experienced long-term SA (>90 days) during the follow-up compared to 7% in the general population. This difference translates to an HR of 4.3 for individuals diagnosed with ADHD in the crude model (Table 2). The risk estimates for long-term SA did not decrease after adjusting for socio-demographic factors but substantially decreased after adjusting for work-related factors (24% lower risk estimate) and comorbid disorders (an additional 13% lower risk estimate). In the final model the risk of long-term SA was thus nearly three times higher in young adults with ADHD than in the matched comparison group (HR = 2.7, Table 2). Sex-stratified analyses showed that young adult men with ADHD had higher risk estimates of long-term SA (HR = 3.0) than young adult women with ADHD (HR = 2.5). The risk estimates of long-term SA were slightly lower compared to the siblings (HR = 2.5, Table 3).

Some 19% of young adults diagnosed with ADHD were long-term unemployed during the follow-up compared to 13% in the general population. In the crude model this equals a 70% higher risk of long-term unemployment (HR = 1.7) compared to the matched comparison group (Table 2). The risk estimates were slightly altered when adjusting for the covariates and the risk of long-term unemployment was still about 70% higher in young adults with ADHD compared to the comparison group in the final model (HR = 1.7, Table 2). In contrast, the risk estimates for long-term unemployment were slightly higher compared to the siblings (HR = 1.9, Table 3). The differences between men and women were small and insignificant.

Concerning the combined measure (i.e. total LMM), young adults diagnosed with ADHD had almost a four times higher risk in the crude model (HR = 3.6) compared to the matched comparison group (Table 2). The risk estimates for the combined LMM decreased when adjusting for work-related factors (8% lower risk estimate) and comorbid disorders (an additional 16% lower risk estimate). In the final model young adults diagnosed with ADHD had nearly three times the risk compared to those in the matched comparison group (HR = 2.7, Table 2). The risk estimates were slightly higher for the siblings (HR = 2.8, Table 3) and the difference in risk estimates between men and women was trivial and not significant.

When excluding specific mental comorbidities, other mental disorders (HR = 14.5, Table 4), autism spectrum disorders (HR = 14.6), behavioural/emotional disorders (HR = 14.6) and mental retardation (HR = 14.6) showed lowest risk estimates for disability pension. For long-term SA, anxiety- and stress-related disorders (HR = 3.1) and depression/bipolar disorders (HR = 3.1) had the lowest risk estimates. Comorbid mental disorders were of less importance for long-term unemployment. The somatic comorbidities, which had the lowest risk estimates for granting disability pension, were asthma (HR = 14.6, Table 4) and neoplasms (HR = 14.6). Somatic comorbidities were of little importance for long-term SA and long-term unemployment.

**Table 4.** HRs with 95% CIs for disability pension, long-term SA (>90 days), long-term unemployment (>180 days) and total LMM (the reference group was the general population) when persons with different comorbid disorders were excluded and compared to persons with diagnosed ADHD (*n* = 9718)

	Disability pension	Long-term SA	Long-term unemployment	Total LMM^a^
	HR (95% CI)	HR (95% CI)	HR (95% CI)	HR (95% CI)
Without excluding any disorder	14.53 (13.34–15.84)	3.24 (3.07–3.43)	1.76 (1.67–1.86)	3.27 (3.15–3.38)
Mental disorders				
Anxiety- and stress-related disorders	15.16 (13.76–16.71)	3.07 (2.88–3.28)	1.79 (1.69–1.90)	3.10 (2.99–3.23)
Autism-spectrum disorder	14.60 (13.38–15.92)	3.24 (3.06–3.43)	1.77 (1.67–1.87)	3.26 (3.15–3.38)
Behavioural/ Emotional disorders	14.56 (13.35–15.87)	3.26 (3.08–3.45)	1.77 (1.67–1.87)	3.28 (3.16–3.40)
Depression/Bipolar disorders	15.21 (13.84–16.70)	3.14 (2.95–3.34)	1.82 (1.72–1.93)	3.19 (3.07–3.32)
Eating disorder	14.51 (13.31–15.82)	3.23 (3.05–3.42)	1.77 (1.67–1.87)	3.26 (3.15–3.38)
Mental retardation	14.56 (13.36–15.87)	3.25 (3.07–3.44)	1.77 (1.67–1.86)	3.27 (3.15–3.39)
Schizophrenia/Psychoses	14.82 (13.58–16.18)	3.25 (3.07–3.44)	1.76 (1.67–1.86)	3.26 (3.14–3.38)
Substance use	14.85 (13.55–16.26)	3.30 (3.11–3.50)	1.74 (1.64–1.85)	3.26 (3.13–3.38)
Other mental disorders	14.57 (13.31–15.95)	3.16 (2.98–3.35)	1.78 (1.69–1.89)	3.20 (3.08–3.32)
Somatic disorders				
Musculoskeletal disorders	14.95 (13.68–16.33)	3.27 (3.08–3.47)	1.78 (1.68–1.88)	3.28 (3.17–3.41)
Asthma	14.61 (13.41–15.93)	3.25 (3.07–3.44)	1.77 (1.68–1.87)	3.27 (3.16–3.39)
Diabetes mellitus	14.76 (13.55–16.10)	3.26 (3.08–3.45)	1.77 (1.67–1.86)	3.28 (3.16–3.39)
Neoplasm	14.57 (13.36–15.88)	3.25 (3.07–3.44)	1.76 (1.67–1.86)	3.26 (3.15–3.38)
Cardiovascular disorders	14.67 (13.46–16.00)	3.26 (3.08–3.45)	1.76 (1.67–1.86)	3.27 (3.15–3.38)
Accidents	15.15 (13.83–16.60)	3.24 (3.05–3.45)	1.80 (1.70–1.90)	3.30 (3.18–3.43)
Other somatic disorders	16.86 (15.11–18.80)	3.44 (3.20–3.70)	1.84 (1.72–1.96)	3.39 (3.24–3.54)

a Adjusted for sex, age and educational level by matching and family composition, type of living area and region of birth, baseline unemployment and baseline SA.

## Discussion

Young adults who were diagnosed with ADHD had a nearly three-fold higher risk for the combined measure of LMM, in which there was an almost 10-fold higher risk of being granted disability pension, about a three-fold higher risk of long-term SA and a 70% higher risk of long-term unemployment compared to the matched comparison group of the same age without ADHD. Comorbid disorders were attributed to about one-third of the association between ADHD and disability pension, but much less for SA and long-term unemployment. Of these comorbidities, autism-spectrum disorders, behavioural/emotional disorders, and mental retardation were key determinants for subsequent disability pension. Depression/bipolar disorders and anxiety- and stress-related disorders were leading risk factors for subsequent long-term SA. Comorbid disorders were less likely to affect long-term unemployment.

The results of this study show that the total burden of ADHD (measured as LMM) was exceedingly high. Two other Swedish longitudinal studies also reported high levels of both disability pension and work absence in adults diagnosed with ADHD (Edvinsson & Ekselius, 2018; Virtanen et al., 2020). In addition, several international cross-sectional studies reported that, for various reasons, work absence seems to be high in patients with ADHD (de Graaf et al., 2008; Fredriksen et al., 2014; Halmoy, Fasmer, Gillberg, & Haavik, 2009; Kupper et al., 2012). As compared to earlier research on ADHD and work-related outcomes, our study was the first that had a broad focus on work-related outcomes. The population-based design and the use of administrative data make our findings robust compared to earlier studies, which were mostly based on small samples and self-reported data. In this study measures based on medical decisions and unemployment were included. The reason for including all the measures is that Sweden and other Nordic countries have a generous social insurance scheme. This scheme is a positive phenomenon that prevents many individuals with functional disabilities facing a financial crisis. Of note, this study revealed a pattern in which the increased risk of long-term unemployment in persons diagnosed with ADHD was just slightly higher compared to the general population. Instead, the risk of work disability (in particular, disability pension) was remarkably high. This outcome was further reinforced as approximately one-fourth of all individuals diagnosed with ADHD during 2006–2011 were awarded a disability pension already before the diagnosis of ADHD and were thus excluded from this study. In countries without welfare contingencies these individuals are at risk of both poverty and declining health. Many young adults in Sweden with mainly mental disabilities have been granted a time-restricted disability pension in young adulthood (Westerholm et al., 2015). For 9 of 10 individuals, this temporary disability pension was transformed into a permanent disability pension at the age of 30 years (Social Insurance Agency, 2012). Authorisation of a disability pension at the age of 19 may thus be the start of lifelong welfare dependence and marginalisation, which might be even more detrimental to the health of an individual as confirmed in several studies (McKee-Ryan et al., 2005; Paul & Moser, 2009). This has hence the potential of a major deterioration in public health. Therefore, the recommendations and policies for work rehabilitation among individuals with ADHD might need revision.

Comorbid mental and somatic disorders are reported to be common in individuals diagnosed with ADHD (Edvinsson et al., 2013; Halmoy et al., 2009). Our results were no exception, showing a 10 times higher prevalence of comorbid mental disorders in individuals diagnosed with ADHD than in the matched comparison group. However, comorbid disorders only contributed to a small fraction of the association between ADHD and the measures of LMM. A study on adults diagnosed with ADHD during childhood reported that those with comorbid disorders had a 60% higher risk of work disability, i.e. disability pension and SA compared to a reference group without such disorders (Virtanen et al., 2020). Thus, comorbid disorders explained to a lesser extent the higher rate of days of disability pension and SA in the current study. One explanation for this discrepancy could be that persons in our study were diagnosed during adulthood *v.* during childhood in the other study and thus our participants may have had less severe symptoms during childhood. Another explanation could be that the measures are not comparable, as that study measured the total amount of days on work disability, whereas our study employed a dichotomised variable. One might conclude that both mental and somatic comorbidities play some role in explaining the high risk of LMM in persons diagnosed with ADHD. However, after considering comorbidities in our study, the risk of LMM was still extremely high. Accordingly, the symptomatic picture of ADHD is responsible for the problematic situation regarding work in young adults diagnosed with ADHD. Of the comorbidities, depression/bipolar disorders, and anxiety- and stress-related disorders had the highest impact on long-term SA. The most important comorbid disorders authorising disability pensions were comorbid autism-spectrum disorders, behavioural/emotional disorders, and mental retardation. Other studies confirm that individuals with these disorders have a high risk of work disability (Helgesson et al., 2017, 2018; McEvilly, Wicks, & Dalman, 2015; Virtanen et al., 2020).

Still, we found a substantially higher risk of LMM in individuals with ADHD compared to their siblings; however, these estimates were lower than when compared to the matched controls from the general population. This finding indicates that a part of the high risk of LMM could be attributed to familial factors. The same pattern was also seen in a study on persons diagnosed with obsessive−compulsive disorders (Pérez-Vigil, Mittendorfer-Rutz, Helgesson, Fernández de la Cruz, & Mataix-Cols, 2018). In general, the role of familial factors has also been studied in twins for SA and disability pension, regardless of diagnosis. Here, heredity did explain parts of the relationship with later work disability (Svedberg et al., 2011). Thus, one additional contribution of the current study is that familial factors were of some importance, but the risk of LMM in young adults with ADHD was still high compared to their siblings.

A few studies have reported sex differences in the symptomatic expression of ADHD (Gershon & Gershon, 2002). In our study we found only small sex differences in LMM, in which only long-term sickness absence had a more substantial difference in the risk estimates. This result may be an indication of that the more internalising problems seen in women might only affect their propensity to be on long-term SA (Edvinsson et al., 2013; Gershon & Gershon, 2002). Even if the symptoms seem different between women and men (Edvinsson et al., 2013), we conclude that men and women diagnosed with ADHD have equal risk estimates for LMM.

### Strengths and limitations

The major strengths of this study include the high quality and completeness of register data that allow individual information on many covariates over a long period. This strength includes the advantage of little loss to follow-up. The inclusion of several measures of LMM is another strength. The large population-based study also provides the possibility of investigating many comorbid disorders. Depending on welfare regime, the scope of LMM might vary between countries. Therefore, a combined measure of LMM, that captures both unemployment and work disability, was introduced. This combined measure may be comparable with the findings from other countries. Limitations of the study also warrant discussion. Both ADHD and all comorbid disorders have been measured by the information available in specialised health care, which most likely represents young adults with medical conditions of greater severity. This fact means that information on less severe conditions has not been covered. Still, estimates on ADHD might not have been strongly affected, as such patients are predominantly treated in specialised health care settings. In our study only persons diagnosed in young adult age were included. Because ADHD cannot be acquired during the life course, the population in this study may have less severe symptoms of ADHD than persons diagnosed during childhood. Moreover, the measure of SA does not include information during the first 14 days of sick leave for employees. Furthermore, information on individuals who are unemployed but who are not registered at The Swedish Employment Agency is not covered in the available dataset. Still, because our outcome variables comprise long-term measures, we are confident that this lack of information does not have a significant effect on the estimates. Finally, despite the availability of a large number of covariates, some information on e.g. body mass index was not available in the registers.

## Conclusions

Young adults with ADHD have a high risk of LMM. Of clinical importance is that comorbidity with other disorders does not play a major role in the association between ADHD and LMM. Regardless of comorbidities, establishment of early interventions targeting work capacity in young individuals with ADHD might be a clinical important intervention to prevent long-term marginalisation of these individuals.

## References

[ref1] Aduen, P. A., Kofler, M. J., Sarver, D. E., Wells, E. L., Soto, E. F., & Cox, D. J. (2018). ADHD, depression, and motor vehicle crashes: A prospective cohort study of continuously-monitored, real-world driving. Journal of Psychiatric Research, 101, 42–49. doi: 10.1016/j.jpsychires.2018.02.026. 29547761 PMC5889746

[ref2] Andersen, P. K., Geskus, R. B., de Witte, T., & Putter, H. (2012). Competing risks in epidemiology: Possibilities and pitfalls. International Journal of Epidemiology, 41(3), 861–870. doi: 10.1093/ije/dyr213. 22253319 PMC3396320

[ref3] Bjorkenstam, E., Pierce, M., Bjorkenstam, C., Dalman, C., & Kosidou, K. (2020). Attention deficit/hyperactivity disorder and risk for non-affective psychotic disorder: The role of ADHD medication and comorbidity, and sibling comparison. Schizophrenia Research, 218, 124–130. doi: 10.1016/j.schres.2020.01.021. 32001080

[ref4] Chen, H.-J., Lee, Y.-J., Yeh, G. C., & Lin, H.-C. (2013). Association of attention-deficit/hyperactivity disorder with diabetes: A population-based study. Pediatric Research, 73, 492. doi: 10.1038/pr.2013.5. 23329200

[ref5] Cortese, S., Sun, S., Zhang, J., Sharma, E., Chang, Z., Kuja-Halkola, R., … Faraone, S. V. (2018). Association between attention deficit hyperactivity disorder and asthma: A systematic review and meta-analysis and a Swedish population-based study. The Lancet Psychiatry, 5(9), 717–726. doi: 10.1016/S2215-0366(18)30224-4. 30054261

[ref6] de Graaf, R., Kessler, R. C., Fayyad, J., ten Have, M., Alonso, J., Angermeyer, M., … Posada-Villa, J. (2008). The prevalence and effects of adult attention-deficit/hyperactivity disorder (ADHD) on the performance of workers: Results from the WHO World Mental Health Survey Initiative. Occupational and Environmental Medicine, 65(12), 835–842. doi: 10.1136/oem.2007.038448. 18505771 PMC2665789

[ref7] Edvinsson, D. (2017). Attention Deficit/Hyperactivity Disorder in Adults - Prevalence, Psychiatric Comorbidities and Long-term Outcome. Thesis. (Thesis). Uppsala University, Uppsala, Sweden.

[ref8] Edvinsson, D., & Ekselius, L. (2018). Six-year outcome in subjects diagnosed with attention-deficit/hyperactivity disorder as adults. European Archives of Psychiatry and Clinical Neuroscience, 268(4), 337–347. doi: 10.1007/s00406-017-0850-6. 29143159 PMC5956008

[ref9] Edvinsson, D., Lindstrom, E., Bingefors, K., Lewander, T., & Ekselius, L. (2013). Gender differences of axis I and II comorbidity in subjects diagnosed with attention-deficit hyperactivity disorder as adults. Acta Neuropsychiatrica. Officieel Wetenschappelijk Orgaan van Het IGBP *(*Interdisciplinair Genootschap voor Biologische Psychiatrie*)*, 25(3), 165–174. doi: 10.1111/j.1601-5215.2012.00682.x. 25287470

[ref10] Fredriksen, M., Dahl, A. A., Martinsen, E. W., Klungsoyr, O., Faraone, S. V., & Peleikis, D. E. (2014). Childhood and persistent ADHD symptoms associated with educational failure and long-term occupational disability in adult ADHD. Attention Deficit and Hyperactivity Disorders, 6(2), 87–99. doi: 10.1007/s12402-014-0126-1. 24497125 PMC4033786

[ref11] Gershon, J., & Gershon, J. (2002). A meta-analytic review of gender differences in ADHD. Journal of Attention Disorders, 5(3), 143–154. doi: 10.1177/108705470200500302. 11911007

[ref12] Giacobini, M., Medin, E., Ahnemark, E., Russo, L. J., & Carlqvist, P. (2018). Prevalence, patient characteristics, and pharmacological treatment of children, adolescents, and adults diagnosed with ADHD in Sweden. Journal of Attention Disorders, 22(1), 3–13. doi: 10.1177/1087054714554617. 25376193

[ref13] Halmoy, A., Fasmer, O. B., Gillberg, C., & Haavik, J. (2009). Occupational outcome in adult ADHD: Impact of symptom profile, comorbid psychiatric problems, and treatment: A cross-sectional study of 414 clinically diagnosed adult ADHD patients. Journal of Attention Disorders, 13(2), 175–187. doi: 10.1177/1087054708329777. 19372500

[ref14] Helgesson, M., Tinghog, P., Niederkrotenthaler, T., Saboonchi, F., & Mittendorfer-Rutz, E. (2017). Labour-market marginalisation after mental disorders among young natives and immigrants living in Sweden. BMC Public Health, 17(1), 593. doi: 10.1186/s12889-017-4504-4. 28645250 PMC5481931

[ref15] Helgesson, M., Tinghog, P., Wang, M., Rahman, S., Saboonchi, F., & Mittendorfer-Rutz, E. (2018). Trajectories of work disability and unemployment among young adults with common mental disorders. BMC Public Health, 18(1), 1228. doi: 10.1186/s12889-018-6141-y. 30400785 PMC6219052

[ref16] Hirvikoski, T., Lindström, T., Nordin, V., Jonsson, U., & Bölte, S. (2017). Föräldraskapsinsatser för föräldrar med ADHD: kartläggning av aktuellt kunskapsläge som grund för utformning av anpassad insats (Parenting initiatives for parents with ADHD: mapping of the current state of knowledge as a basis for designing adapted initiatives) [In Swedish]. (Vol. 2017:3): Linnéuniversitetet.

[ref17] Janlert, U., & Hammarstrom, A. (2009). Which theory is best? Explanatory models of the relationship between unemployment and health. BMC Public Health, 9, 235. doi: 10.1186/1471-2458-9-235. 19602230 PMC2720386

[ref18] Koller, M. T., Raatz, H., Steyerberg, E. W., & Wolbers, M. (2012). Competing risks and the clinical community: Irrelevance or ignorance? Statistics in Medicine, 31(11–12), 1089–1097. doi: 10.1002/sim.4384. 21953401 PMC3575691

[ref19] Kupper, T., Haavik, J., Drexler, H., Ramos-Quiroga, J. A., Wermelskirchen, D., Prutz, C., & Schauble, B. (2012). The negative impact of attention-deficit/hyperactivity disorder on occupational health in adults and adolescents. International Archives of Occupational and Environmental Health, 85(8), 837–847. doi: 10.1007/s00420-012-0794-0. 22752312

[ref20] Lehti, V., Chudal, R., Suominen, A., Gissler, M., & Sourander, A. (2016). Association between immigrant background and ADHD: A nationwide population-based case-control study. Journal of Child Psychology and Psychiatry and Allied Disciplines, 57(8), 967–975. doi: 10.1111/jcpp.12570. 27133554

[ref21] McEvilly, M., Wicks, S., & Dalman, C. (2015). Sick leave and work participation among parents of children with autism spectrum disorder in the Stockholm Youth Cohort: A register linkage study in Stockholm, Sweden. Journal of Autism and Developmental Disorders, 45(7), 2157–2167. doi: 10.1007/s10803-015-2381-1. 25697737 PMC4471388

[ref22] McKee-Ryan, F., Song, Z., Wanberg, C. R., & Kinicki, A. J. (2005). Psychological and physical well-being during unemployment: A meta-analytic study. Journal of Applied Psychology, 90(1), 53–76. doi: 10.1037/0021-9010.90.1.53. 15641890

[ref23] Nilsson, S. (2017). Employability, employment and the establishment of higher education graduates in the labour market. In M. Tomlinson & L. Holmes (Eds.), Graduate employability in context (pp. 65–85). London: Palgrave Macmillan.

[ref24] Paul, K. I., & Moser, K. (2009). Unemployment impairs mental health: Meta-analyses. Journal of Vocational Behavior, 74(3), 264–282. doi: 10.1016/j.jvb.2009.01.001.

[ref25] Pérez-Vigil, A., Mittendorfer-Rutz, E., Helgesson, M., Fernández de la Cruz, L., & Mataix-Cols, D. (2018). Labour market marginalisation in obsessive–compulsive disorder: A nationwide register-based sibling control study. Psychological Medicine, 49(6), 1015–1024. 29950186 10.1017/S0033291718001691

[ref26] Rydell, M., Lundström, S., Gillberg, C., Lichtenstein, P., & Larsson, H. (2018). Has the attention deficit hyperactivity disorder phenotype become more common in children between 2004 and 2014? Trends over 10 years from a Swedish general population sample. Journal of Child Psychology and Psychiatry, 59(8), 863–871. doi: 10.1111/jcpp.12882. 29484650

[ref27] Social Insurance Agency (2012). Tio år med aktivitetsersättning- en studie av situationen för unga med aktivitetsersättning på grund av nedsatt arbetsförmåga (Ten years with activity compensation − a study on the situation among young adults with activity compensation due to work disability) [In Swedish]. Retrieved from.

[ref28] Svedberg, P., Narusyte, J., Samuelsson, Å., Ropponen, A., Lichtenstein, P., & Alexanderson, K. (2011). Betydelsen av arv och miljö för sjukskrivning och sjukersättning bland kvinnor och män i en kohort av svenska tvillingar (The importance of heredity and environment for sick leave and sick pay of women and men in a cohort of Swedish twins [In Swedish] Karolinska Institutet.

[ref29] Thomas, R., Sanders, S., Doust, J., Beller, E., & Glasziou, P. (2015). Prevalence of attention-deficit/hyperactivity disorder: A systematic review and meta-analysis. Pediatrics, 135(4), 994–1001. doi: 10.1542/peds.2014-3482. 25733754

[ref30] Virtanen, M., Lallukka, T., Kivimaki, M., Alexanderson, K., Ervasti, J., & Mittendorfer-Rutz, E. (2020). Neurodevelopmental disorders among young adults and the risk of sickness absence and disability pension: A nationwide register linkage study. Scandinavian Journal of Work, Environment and Health, 46(4), 410–416. doi: 10.5271/sjweh.3888. PMC8506319 32076730

[ref31] Westerholm, P., Lundberg, I., Anderzén, I., Lytsy, P., Helgesson, M., Gustafsson, M., & Palmer, E. (2015). Evidence-based methods for enhancing the labour force entrance of people with mental disabilities − A systematic literature review. Retrieved from Stockholm:.

[ref32] Wiklund, J., Patzelt, H., & Dimov, D. (2016). Entrepreneurship and psychological disorders: How ADHD can be productively harnessed. Journal of Business Venturing Insights, 6, 14–20. doi: 10.1016/j.jbvi.2016.07.001.

